# Rethinking the methodology of global indexes for equitable evidence-informed policy towards sustainable development

**DOI:** 10.7189/jogh.14.03047

**Published:** 2024-12-20

**Authors:** Kehinde O Ogunyemi, Chima Ohuabunwo, Virgil K Lokossou, Lionel S Sogbossi, Simon Antara, Tolbert Nyenswah, Melchoir A Aïssi, Scott McNabb, Senait Kebede

**Affiliations:** 1 Hubert Department of Global Health, Rollins School of Public Health, Emory University, Atlanta, Georgia, USA; 2 Morehouse School of Medicine, Department of Community Health and Preventive Medicine, David Satcher Global Health Equity Institute, Atlanta, Georgia, USA; 3 ECOWAS Regional Centre for Surveillance and Disease Control, West African Health Organization, Bobo Dioulasso, Burkina Faso; 4 African Field Epidemiology Network, Kampala, Uganda; 5 Department of International Health, Bloomberg School of Public Health, Johns Hopkins University, Baltimore, Maryland, USA; 6 Emory University, Rollins School of Public Health, Emory Global Health Institute, Atlanta, Georgia, USA

Rethinking the methodology of global indexes (e.g. multidimensional poverty index (MPI), global health security index (GHSI), and human development index (HDI)) to inform effective and equitable policy actions for improved outcomes at the population and system levels towards charting the path to sustainable development has never been more important than now due to increasing health and socioeconomic losses from the past, present and future global problems [1–4]. These problems comprise the past coronavirus disease 2019 (COVID-19) pandemic, the present increasing impact of climate change, and the future risk of world population explosion that threatens the sustainability of the human-animal-environmental ecosystem, and the development of countries, particularly in low- and middle-income countries (LMICs), where these issues are disproportionately substantial [5–10]. For LMICs, the gross domestic product (GDP) per capita in 2024 is projected to be 6% lower than the level expected before the pandemic compared to 2% in high-income countries (HICs) [5]. Further, not only is this economic disparity likely to be worse in LMICs – where conflict and violence are frequent [6,7], it has also been reported that LMICs would bear more than two-thirds of the global labour productivity loss due to excessive heat from climate change that contributes to an unfavourable working condition for its population [8,9]. More so, the uncertainty level for development in LMICs is significant given the increased likelihood of high demand for public goods and services from its growing population and their huge vulnerability to poor governance [10], which may limit the ability of governments to provide effective policies for efficient, equitable, and sustainable use of scarce resources to improve key outcomes (e.g. health, social, economic) and reform the systems that shape them.

In addition to the high burden of debt in LMICs [11,12], these global issues have the potential to cause further widening of the existing economic divide or create new ones and may increasingly constrain the implementation of appropriate policies such as a fiscal policy that increases proportionate taxation and optimal spending on public goods and services (e.g. social protection, health, education) and systemic interventions that enhance governance to improve the development outcomes (e.g. poverty reduction, health security, human development) that are measured by many global indexes [13,14].

While global indexes provide essential information on the performance of countries regarding specific outcomes to inform appropriate policy and systemic interventions and investments, several gaps and limitations, plus related ethical problems in their conceptualisation, analysis, and presentation, have been reported in the literature. These fall into three major categories, including inadequate stakeholder representation and transparency in the conceptualisation of global indexes that weakens internal validity; the risk of biased results due to inconsistent and incomplete data across countries that threaten external validity; and limited cross-country comparability with an international ranking approach given baseline differences in the context within and between countries as well as ethical issues on fairness, inclusiveness, and the moral commitments to the Sustainable Development Goals (SDGs), which all constrain our ability to draw meaningful interpretations and conclusions for necessary actions [15–22].

**Figure:**
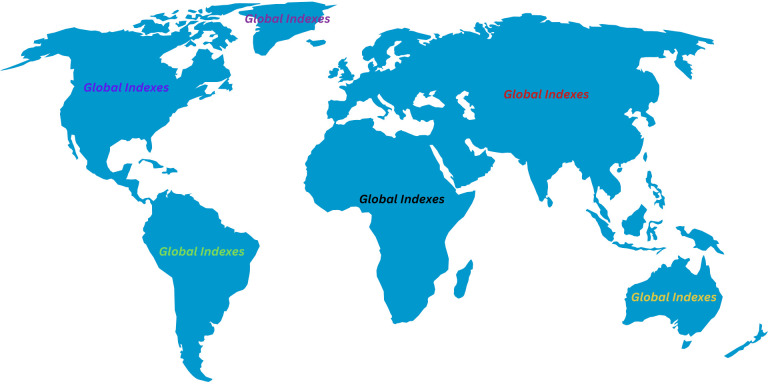
**Photo:** World Map Earth Global (edited). Source: Pixabay. In public domain (free for use under the Pixabay Content License). Available at: https://pixabay.com/vectors/world-map-earth-global-continents-306338/.

## GAPS AND LIMITATIONS IN THE CURRENT METHODOLOGY OF GLOBAL INDEXES

The development of global indexes is rapidly increasing (**Table 1**), yet there is a growing concern about the gaps and limitations in the current methodology used in their conceptualisation, analysis, and presentation that are worthy of greater attention and timely change actions from global institutions and political actors (**Table 2**). First, many have argued that the development processes of global indexes deviate from the principle of collective action that encourages diversity, inclusion, and equity. It has been widely recognised that there is a need to ensure that the processes used in their conceptualisation should be more representative (i.e. inclusive of LMICs stakeholders with equitable decision-making opportunities) and transparent (i.e. providing open-access information on the definitions and rationales of the need, significance, selected variables, weights attributed to the selected variables, and assumptions for external validation and peer review) to improve their internal validity [16–21]. For instance, in a 2019 scoping review conducted by Ashraf and colleagues, it was reported that of the 27 population health indexes identified, 52% had at least one aspect of their development processes documented. The same study also indicated that only 26% of these indexes were developed based on an a priori theoretical or empirical framework that is fundamental to making them scientifically sound and accurate [21].

**Table 1:** Characteristics of common examples of global indexes widely recognised for SDGs [2,4,23–26]

Characteristics	MPI	GHSI	HDI
Innovator/adopter	Oxford Poverty & Human Development Initiative and the United Nations Development Program	Nuclear Threat Initiative, Johns Hopkins University Centre for Health Security, and the Economist Intelligence Unit	United Nations Development Program
Release year	2010	2019	1990
Reporting period	Annually	2019 and 2021 till date	Annually
Outcome	Assess a country’s level of poverty by calculating poverty incidence (i.e. the average percentage of people who experience multiple deprivations or are poor) and intensity (i.e. the average percentage of deprivations experienced by the poor).	Assess a country’s level of global health security (i.e. the state of preparedness with technical, socioeconomic, and political capabilities of a country to prevent, detect, and respond to public health threats or events with the risk of international spread).	Assess a country’s level of human development.
Dimension	Three dimensions. Health, education, and standard of living.	Six dimensions. Prevention, detection and reporting, rapid response, health system, compliance with international norms, and risk environment.	Three dimensions. Health, knowledge, and standard of living.
Indicator	10 indicators. Health: nutrition and child mortality. Education: years of schooling, school attendance. Standard of living: cooking fuel, sanitation, drinking water, housing, electricity, and assets.	34 indicators. Prevention: antimicrobial resistance, zoonotic disease, biosecurity, biosafety, dual-use research and culture of responsible science, and immunisation. Detection and reporting: laboratory systems strength and quality, laboratory supply chains, real-time surveillance and reporting, surveillance data accessibility and transparency, case-based investigation, and epidemiology workforce. Rapid response: emergency preparedness and response planning, exercising response plans, emergency response operations, linking public health and security authorities, access to communication infrastructure, and trade and travel restrictions. Health systems: health capacity in clinics, hospitals, and community care centres, supply chain for health system and health care workers, medical countermeasures and personnel deployment, health care access, communications with health care workers during a public health emergency, infection control practices, and capacity to test and approve new medical countermeasures. Compliance with international norms: IHR reporting compliance and disaster risk reduction, cross-border agreements on public and health emergency response, international commitments, JEE and PVS, financing, and commitment to sharing of genetic and biological data and specimen. Risk environment: political and security risk, socio-economic resilience, infrastructure adequacy, environmental risks, and public health vulnerabilities.	Three indicators. Health: life expectancy at birth. Knowledge: expected years of schooling and mean years of schooling. Standard of living: gross national income per capita.
Applicable setting	LMICs	HICs and LMICs	HICs and LMICs
Country coverage	110 countries	195 countries	193 countries
Ranking scoring	Descending order statistics on a 0–1 scale	66.7–100 (high), 33.4–66.6 (moderate), 0–33.3 (low)	0.800–1 (very high), 0.700–0.799 (high), 0.550–0.699 (medium), 0–0.540 (low)

GHSI – global health security index, HDI – human development index, HICs – high-income countries, IHR – International Health Regulations, JEE – Joint External Evaluation, LMICs – low- and middle-income countries, MPI – multidimensional poverty index, PVS – Performance of Veterinary Services

**Table 2:** Gaps and limitations in the conventional methodology of some global indexes

Gap	MPI	GHSI	HDI	Limitations	
Conceptualisation*	Evidence suggests that several experts were consulted in its development [4,27,28], but specific details about their selection method (e.g. random and/or non-random), experience (e.g. technical and/or lived), and country (e.g. HICs and/or LMICs) are lacking. Evidence indicates its endorsement by several experts [4,27,28], but the findings of its validation conceptually (e.g. the aptness of indicators based on an a priori theoretical or empirical framework, indicators cut-offs justification) and statistically using widely known tests (e.g. reliability, validity) were not reported. Although, it was adopted based on a methodology developed by Alkire and Foster that demonstrated how poverty incidence and intensity could be calculated from a set of selected indicators and dimensions [4]. Still, the methodology did not clearly describe the link between the selected indicators and poverty – a limitation acknowledged by the authors given their recommendation for additional normative criteria in the selection of indicators [4].	21 experts from 13 countries including HICs and LMICs were reported to have supported its development [25,26,29], but specific information about their selection method is unknown. Also, the findings of its validation conceptually (e.g. the aptness of indicators based on an a priori theoretical or empirical framework, indicators cut-offs justification) and statistically using widely known tests (e.g. reliability, validity) are lacking.	Evidence indicates that other experts supported its development following its conceptualization by Mahbub ul-Haq in collaboration with Amartya Sen [24,28,30]. However, specific details about their selection method, experience, and country are lacking. Further, the findings of its validation conceptually (e.g. the aptness of indicators based on an a priori theoretical or empirical framework, indicators cut-offs justification) and statistically using widely known tests (e.g. reliability, validity) are lacking are unavailable.	There is an increased likelihood of unbalanced judgments due to inadequate representation of experts. The risk of confirmation bias is high given the lack of a clear theoretical framework to make indicators and assumptions falsifiable. Their internal validity and internal consistency reliability remain unknown due to a lack of conceptual and statistical validation based on widely recognised scientific methods.	
**Analysis**†	Different sources of population-level microdata (e.g. DHS, MICS, national surveys) are used based on what is available in each country, which may not be collected at the same time (e.g. every five years for DHS, every three years for MICS) [27,28,31]. Additionally, some dimensions data are often unavailable and the justification for the missing data handling (e.g. imputation, weight readjustment) is lacking.	Different sources of system-level aggregate data (e.g. government reports, literature, WHO documents) are used based on what is available in each country, which may not be collected at the same time [25,26,29]. More so, data for all dimensions are often unavailable or unverifiable (e.g. 19 of 195 countries were verified) [25] and lacks missing data handling details.	Different sources of population-level aggregate data (e.g. DHS, MICS, UNDESA documents) are used based on what is available in each country, which may not be collected at the same time [24,28,30]. Also, dimensions data are often unavailable and the justification for the missing data handling is lacking.		There is a higher risk of selection bias due to potential differences in the methods and time point of data collection. They are prone to information bias due to data scarcity/missingness. Their external validity is limited given the possible systematic differences in the observed and missing.
**Presentation**‡	Disaggregated by its independent variables (e.g. age, locality) [27]. However, it lacks disaggregation by contextual factors (e.g. debt, foreign investment, or assistance). Also, countries are grouped in descending order of value at the global and national levels but not at the regional level.	Lacks disaggregation by contextual factors [29]. Further, countries are ranked at the global level, but not at the regional and national levels.	Disaggregated by its independent variables [24]. However, it lacks disaggregation by contextual factors. More so, countries are ranked at the global and regional levels, but not at the national level.		There is an increased likelihood of interpretation bias due to a lack of contextual information underlying results. The international ranking of countries’ indexes is constrained because of differences in context.

DHS – demographic and health survey, GHSI – global health security index, HDI – human development index, HICs – high-income countries, LMICs – low- and middle-income countries, MICS – multiple indicator cluster surveys, MPI – multidimensional poverty index, UN – United Nations, UNDESA – United Nations Department of Economic and Social Affairs. WHO – World Health Organization

*Representativeness: diversity, inclusion, and equity for indexes. Transparency: external validation and peer review of indexes.

†Data quality: consistency of data sources for indexes. Data robustness: completeness of data for indexes.

‡Results granularity: context-specific interpretation of indexes. Comparable ranking: ranking of indexes by country’s level of development.

Second, there are concerns about information bias from inconsistent and incomplete data across indicators and countries and how this may limit their external validity [16–21]. Specifically, it is thought that the indicators of many global indexes may not be accurate nor comparable between countries because, for example, the same indicator may be collected from different publicly available data sources (e.g. national surveys, government reports) with separate time points depending on what is feasible in each country. LMICs such as those in Africa are more likely to lack needed data, recent data, or complete data largely due to limited resources and suboptimal digital infrastructure for conducting high-quality surveys and making data publicly available [32]. This gap can lead to their inadvertent exclusion from important global indexes assessment or use of unrepresentative data because the condition of the country might have changed or make their data prone to biased assumptions during missing data imputation procedures. For example, the 2023 MPI report for 110 countries showed that Bhutan, Burkina Faso, and South Sudan were excluded from the assessment because they lacked recent data [23]. The same report also revealed National Demographic Health Surveys (DHS) (43 countries), Multiple Indicator Cluster Surveys (MICS) (54 countries), Pan Arab Project for Family Health surveys (two countries), and national surveys (11 countries) as its primary data sources with time points that varied between 2011 and 2021–22 [23]. Deficiencies like this make cross-country comparability even more difficult, in addition to the inherent differences in the validity of indexes for each country. This assumption is based on the general understanding that national surveys with microdata are more likely to have a lower level of uncertainty compared to government reports with aggregated data that may have inaccurate or inconsistent data sources. Further, qualitative data are rarely triangulated with quantitative data to provide a more holistic understanding of their results.

Third is the issue around their presentation with an international ranking approach, which has been a subject of criticism over the past 34 years since the release of the popular HDI [2]. Depending on where we choose to stand, no ranking approach is superior to another. It is about finding a middle ground, which will require some trade-offs. From the globalised world point of view, the interconnectedness and interdependence of countries by trade, travel, investment, and assistance, where the action of one country can have a multiplier effect on others, an international ranking can be taken as an accountability measure to monitor and spur countries’ progress through healthy competition towards a common national or global public good like the SDGs [33,34]. On the other hand, there is the philosophical view that this approach shifts people’s focus to just ‘rank’ and carries some negative risks that may undermine its intended purpose of informing ‘policy actions’ [34–36]. This can also be well-pronounced or even overshadow its success stories. First, countries and their entities (policymakers, business owners, and the general public) can become subjects of name shaming when they have low ranks in any global indexes, which may result in a sequential chain of emotions, including resentment, apathy, and inaction following internalisation of their poor performances, especially if their rankings do not improve over time [36]. Second, external actors’ (international governments, investors, non-governmental organisations (NGOs), and donors) cooperation with poorly-rated countries via trade and investment may be greatly influenced in a complex and unpredictable way depending on their interests, priorities, and power (e.g. reduced investment and trade from international governments and businesses, increased assistance from NGOs and donors) [34,36].

In the same light, this practice may put undue pressure on high-performing countries to overinvest in one policy area at the expense of another to sustain their ranks. Thus, it is becoming a counterproductive approach for global institutions (e.g. the United Nations system) in monitoring the progress of countries towards sustainable development or for the higher education system in monitoring the progress of universities towards academic excellence as already being observed worldwide [31,37]. For example, the World Health Organization (WHO) has shifted completely from conducting any form of cross-country ranking with their indexes (e.g. Universal Health Coverage service coverage index, Joint External Evaluation (JEE) tool) to providing countries with scores that can be used as quantitative baselines in supporting them to monitor their own progress towards outcomes like global health security [38,39]. The same parallel can be drawn from the education sector, where many high and low-ranked universities around the world are opting out of the international university ranking system, citing flawed methodology, unnecessary pressure, and implicit agenda as reasons for their exit [31,37].

From a methodological standpoint, several scholars share the view that the disparate socioeconomic losses from the past and emerging global issues between HICs and LMICs stressed above do not justify the current international ranking approach of all global indexes. It is further argued that based on the significant differences in the baseline economic, sociocultural, political, and anthropogenic characteristics of countries [5–10], a combined ranking of both LMICs and HICs could be equated to comparing ‘apples with oranges’ – a phenomenon that completely deviates from the epidemiological principle of comparing ‘like with like.’ Typically, no two countries have had the same experience, and the reasons underlying these differences are complex and difficult to quantify or adjust for while estimating any global indexes.

More so, the justification of an international ranking would be heavily rested on how historical events (e.g. colonialism, wars), international market policies (e.g. interest rates, sanctions), debts (e.g. intergenerational debt, debt relief), foreign investment and assistance (e.g. businesses, aids), and externalities (e.g. violence, epidemics) are accounted for. The inappropriateness of this ranking approach is further supported by evidence that suggests that most global indexes might only show results from the direct effects of countries’ development level – as demonstrated in studies that showed positive correlations of GDP with many global indexes, including HDI [15,16], and also inaccurately capture the true state of outcomes [17,21,22]. While this ranking approach, from a global perspective, is expected to show positive or negative deviations from an assumed performance based on the development level of a country, the regional and national/subnational usefulness of these indexes by policymakers to better understand the outcomes measured, assess capacity, and inform effective and equitable policy actions may become lost due to lack of contextual information other than GDP-related information, which only perpetuates a dominance culture of HICs over LMICs. Hence, fairness is another ethical issue that should be considered. This is vital to ensure that a ranking approach that reflects the true reality of outcomes as much as possible and does not disadvantage one country or group of countries over another is adopted.

### Ethical issues

These gaps and limitations of global indexes have several ethical issues that should be carefully examined and addressed. Data are the backbone of any global index. And as such, its ethical use must be a foundational principle that is promoted and regulated across their conceptualisation, analysis, and presentation. Regrettably, very little attention has been given to the ethical considerations in their design and use, and there is a lack of a common consensus on what should constitute their ethical standards. With the assumption that data for global indexes will be generated from ethically sound nationally-representative research that adhered to the core ethical principles of respect for persons (value and protect people’s opinions and choices), beneficence (maximise benefits and minimise risks), non-maleficence (do no harm), and justice (fairness and equity), the conceptualisation, analysis, and presentation of global indexes also need to be rooted on these principles and guided by the ethical standards in the fields of measurement science and data science [40–43]. Innovators, adopters, and users of global indexes have a responsibility to conceptualise, analyse, and present global indexes based on data ethics (i.e. the ethical obligations in the creation, collection, processing, and dissemination of data) [44].

**Figure 1:**
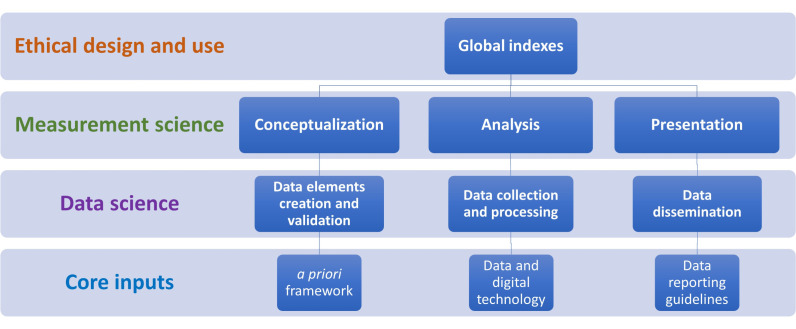
Role of measurement science, data science, and core inputs in achieving the ethical design and use of global indexes.

Data ethics principles, including but not limited to compliance with data privacy, confidentiality, fairness, inclusiveness, transparency, accountability, safety, and security, are crucial for global indexes to maximise the benefits of data for the public good while minimising potential risks (**Figure 1**, **Box 1**) [44–46]. However, evidence of biased results from inadequate inclusion of stakeholders in their conceptualisation, particularly those from underserved settings and exclusion of some countries in their analysis due to unavailability of data, and the use of international ranking that tends to overgeneralise results, plus associated considerable risks (name shaming, reduced international trade and investment) among countries with low ranks are practices that deviate from the principles of data fairness and inclusiveness as well as the moral commitments of global institutions and political actors, including the United Nations (UN), to leave no one behind and support national development as a means to achieving the SDGs. This is because if all relevant stakeholders and countries are included, their results will be more accurate, and countries’ ranks are likely to reflect the true reality of their performance (e.g. a country’s previous ranking may change from ‘low’ to ‘medium’ or vice versa) [33]. Therefore, having strategies in place to mitigate these ethical issues is urgently needed.

Box 1 **Operational definitions of key scientific and ethical terms related to global indexes**

Fields related to the conceptualisation, analysis, and presentation of global indexes

Measurement science – ‘the field of creating critical-solution enabling tools – metrics, models, and knowledge’ [41].

Data science – ‘the interdisciplinary field of inquiry in which quantitative and analytical approaches, processes, and systems are developed and used to extract knowledge and insights from increasingly large and/or complex sets of data’ [43].

Ethical principles for the conceptualisation, analysis, and presentation of global indexes

Data ethics – ‘are the norms of behaviour that promote appropriate judgments and accountability when acquiring, managing, or using data, with the goals of protecting civil liberties, minimising risks to individuals and society, and maximising the public good’ [44].

Data privacy – is freedom from unwarranted intrusion into the private lives of individuals and private conduct of organisational operations (i.e. the protection of an individual’s or organisation’s right to control the way their data are collected, stored, analysed, and disseminated [45].

Data confidentiality – is the state of individual and organisation’s personal information being free from unauthorised access and use (i.e. the protection of personal and organisation data, including identifiable and sensitive information from inappropriate access, disclosure, or theft [45].

Data fairness – ‘is an approach to achieve inclusive representation. It minimises human bias in research and data collection so that all communities are fairly and objectively represented. Fairness also means mitigating bias and ensuring data projects do not result in unintended effects on social groups and individuals’ [45].

Data inclusiveness – ‘means that all relevant people have an equal opportunity to be included in the data collected and in the data’s use and that no one is left out, voices are heard, people have equal access to data, and people can understand the data. Vulnerable communities must be intentionally considered and included’ [45].

Data transparency – ‘is the open disclosure and sharing of information about a project in a complete, clear, intelligible, and easily accessible format’ [45].

Data accountability – ‘is setting and fostering a common expectation by clearly defining the organisation’s mission, values, and goals while acknowledging responsibilities for actions, decisions, and products. Accountability requires that anyone acquiring, managing, or using data be aware of stakeholders and responsible to them, as appropriate’ [45].

Data safety – ‘is protecting data against unintentional loss and restoring data as necessary’ [45].

Data security – ‘is the practice of protecting digital information from unauthorised access, corruption, or theft throughout its lifecycle’ [45].

### Policy and practice implications of rethinking the current methodology of global indexes

The highlighted gaps and limitations in the methodology of global indexes have some policy and practice implications. First is the potential for poor understanding of the outcome (problem or benefit). Until it can be explicitly demonstrated that the outcome definitions, selection of variables (dimensions and indicators), and rationale of assumptions in global indexes that have informed their development are based on sound theoretical underpinnings or empirical frameworks through whole-of-society, collaborative, and equitable processes, the issue of their validity in providing a true understanding of what they intend to measure remain a significant concern that calls for urgent change actions. The second is inadequate priority setting and policy implementation due to potentially inaccurate outcome assessment. With the knowledge that the results of global indexes are supposed to guide policymakers in identifying the outcomes, dimensions, and indicators at the subnational/national, regional, and global levels with limited performances for policy and systemic interventions and investments to address them, the gaps in their data quality could lead to an endless cycle of having wrong dimensions and indicators prioritised for improvement [34]. For every inaccurate global index, there is a missed opportunity for meaningful interventions and investments to yield real-world impact. Third is suboptimal resource mobilisation and allocation for the improvement of people’s lives and the strengthening of national capacities. A flawed estimation of global indexes will limit policymakers from correctly identifying the most poorly performing states/countries or regions and prevent the optimal prioritisation of resources for targeted policy and systemic interventions to improve the dimensions and indicators with inadequacies. Thus, having an index that gives a true estimate of its measures within and between countries is fundamental in effectively mobilising and allocating resources to implement existing or new policies at the subnational/national, regional, and global levels. Fourth is an incremental and inequitable impact. In the longer term, the high tendency of the current methodology of global indexes to yield erroneous results and conclusions could be counterproductive by leading to small changes in health and social outcomes at the population and system levels as well as the widening of existing or creation of new inequities unless urgent and decisive actions are taken to have them refined or reconceptualised. Overall, a radical shift in thinking and practice of all stakeholders is needed. This is particularly important in contributing towards achieving the SDGs by 2030 without leaving anyone behind [33].

## RECOMMENDATIONS

**Figure 2:**
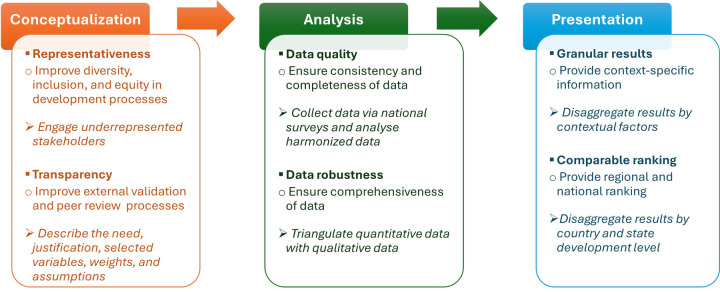
Proposed conceptualisation, analysis, and presentation (CAP) principles and practices for improving the ethical design, use, and impact of global indexes.

To address these gaps and limitations, six key principles, along with their best practices, are recommended for improving the design, use, and impact of global indexes and represented by the acronym CAP principles and practices (i.e. conceptualisation with representativeness and transparency by including underrepresented stakeholders and the description of development processes; analysis of quality data and robust data by using national surveys and mixed-methods design; and presentation of granular results and comparable ranking by disaggregating results by contextual information and country/state development level) (**Figure 2**).

### Conceptualisation

#### Improve representativeness of stakeholders in global indexes development

Sufficient representativeness of stakeholders needs to occur at all stages in the development of global indexes by ensuring extensive engagement of professionals, policymakers, and populations with relevant expertise and experience. From index construction and validation to their adoption and usage by independent bodies (e.g. individuals, groups) or global institutions (e.g. UN Development Programme, WHO), stakeholders should be included in a substantial diversity of expertise (multisectoral, multidisciplinary, multispecialty) and equitable context of experience (government, industry, civil society) for iterative, comprehensive reviews and deliberations. By embracing the complexity of variables (outcomes, dimensions, and indicators) and diversity of perspectives through collaborative engagement with stakeholders, who bring into such deliberations the numerous political, socioeconomic, and ecological complexities facing their countries, there is an increased opportunity for developing more scientifically sound, robust global indexes that capture the outcomes of interest effectively and equitably. Importantly, it is recommended that priority should be given to underrepresented stakeholders (e.g. region: Africa, Middle East, and Asia; sex: women; age group: youths), who are widely known to be historically excluded from many scientific and decision-making efforts to have balanced perspectives reflected in the characterisation of global indexes outcome and the selection and definition of their dimensions and indicators [44,45,47]. More so, it is expected that at least one stakeholder, including a subject matter expert, should be consulted in each country of the applicable setting (HICs, LMICs, or both) during their refinement before their widespread deployment if indeed they are designed for global use and impact. Having the right set of indicators is fundamental for making accurate interpretations and policy actions, and this practice must be ensured, critiqued, and refined as needed.

#### Improve transparency of development processes

Development processes for several global indexes remain unclear [18,19,34]. Their innovators/adopters should provide an open-access report with explicit descriptions of the selection method, nature of experience, and country of stakeholders consulted for their external validation, plus their development approaches, theories, and methods, including a statement of or reference to scholarly peer review, in which the public can regularly review to provide critiques and contributions. In this report, it is also recommended that the need (statement of the outcome), justification (implications of the outcome), selected variables (dimensions and indicators definition and an a priori theoretical or empirical framework), weights (numeric relative importance of dimensions and indicators of an outcome), and normative assumptions (rationale) of such index should be clearly articulated to facilitate ongoing constructive feedback for its continuous refinement based on changing global body of knowledge and context. While global indexes are typically presented as ‘technical frameworks’ by influential institutions and actors that are often motivated by their interests, priorities, and powers, without having detailed information on these characteristics, the notion that most of them may have been conceptualised based on value judgments with little engagement of relevant stakeholders will continue to hold true [33,34]. Thus, reducing their validity in supporting decision-making for critical policy and systemic interventions and investments at the sub-national/national, regional, and global levels. To address these gaps and limitations in existing global indexes, a correlation analysis should be conducted across countries to assess the direction and magnitude of the relationship between their outcomes and dimensions, such that when there is a strong-moderate positive relationship (i.e. both global index and dimension index increase or decrease together), the dimension is said to be a valid construct of the global index as demonstrated with HDI by Yakunina and colleagues [48]. A dimension index can be calculated as the arithmetic mean of the normalised unweighted scores of their indicators at the country level, as frequently provided in GHSI and HDI reports [24,25] and illustrated in **Box 2** for MPI. However, given the variability in the interpretation of the magnitude of a correlation coefficient, a consensus must be reached for its cut-offs. Additionally, because global indexes often contain more than one dimension, their validity could be established with a conservative cut-off when 50% of its dimensions have a strong-moderate positive relationship.

Box 2 **Proposed steps for estimating each dimension index of the multidimensional poverty index (MPI)**

Step 1: Calculate the average number of household members who experienced deprivation in each indicator of an MPI dimension (i.e. a positive response to the indicator question that is assigned a value of 1) for each country.

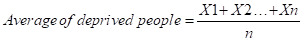
,

where x̄ presents the mean, *X*1 presents the number of deprived people in an indicator in household 1, *X*2 is the number of deprived people in an indicator in household 2, and n is the total number of households.

Step 2: Calculate the normalised score for the average of deprived people in each indicator of an MPI dimension for each country.

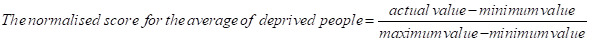
,

Where actual value is the value of the average of deprived people in an indicator in a country, the minimum value is the lowest value of the averages of deprived people in an indicator in all countries, and the maximum value is the lowest value of the averages of deprived people in an indicator in all countries.

Step 3: Calculate each MPI dimension index for each country.

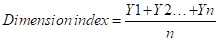
,

where Y1 is the normalised score for the average of deprived people in indicator 1 in a country, Y2 is the normalised score for the average of deprived people in indicator 2 in a country, and n is the total number of dimension’s indicators.

### Analysis

#### Ensure data quality

Given the understanding that a majority of global indexes rely heavily on publicly available data at the national level that are mostly from disparate sources (e.g. MICS, DHS, national surveys), incomplete, and collected with different methods and at varied time points [24,49], a common data source predesigned to capture the variables of global indexes adequately is warranted to ensure the inclusion of all countries and improve data quality for cross-country comparability. This can be achieved by integrating their questionnaires into national surveys that should be ideally collected annually and by ensuring the availability of dedicated pooled domestic and international resources to support data collection, analysis, interpretation, and dissemination. There are five main advantages of leveraging national surveys for collecting data on global indexes. First, it will allow closer collaboration between national governments and innovators/adopters of global indexes to establish consensus in variable definitions, survey methods, and data collection time points. Second, it has concurrent benefits of improving transparency and local capability in data collection, analysis, interpretation, and dissemination. Third, it will provide access to real-world, real-time microdata with improved cross-country comparability for a comprehensive understanding of the population-level or system-level factors that might contribute to geographic variation in the outcomes of interest and their dimensions and indicators nationally, regionally, and globally. Fourth, it will increase local ownership of global indexes and the ability of states and countries to act on the data to target relevant resources for evidence-informed policymaking. Fifth, it will contribute to the reduction of duplicated efforts and provide a cost-saving benefit by reducing personnel recruitment and training costs for data collation from additional surveys such as MICS and DHS.

Similarly, it is recommended that, where possible, data should be harmonised to reflect changing contexts for accurate interpretations and actionable insights. Harmonising data helps to ensure that the results for trends over time are presented after adjusting for changes in the index variables, variable definitions, and data analysis methods. This is a practice that the Oxford Poverty and Human Development Initiative and the UN Development Programme promote for MPI and HDI and should be commended for [2,23].

#### Ensure data robustness

For a comprehensive understanding of the outcomes, dimensions, and indicators of global indexes, it is crucial to increase the robustness of data. Though quantitative data have been the backbone of most global indexes, the role of qualitative data should be given increased recognition and their potential benefits fully capitalised. Qualitative data collected, for example, through key informant interviews with subject matter experts in different countries, could help not only to have an in-depth understanding of the dimensions and indicators and the social and structural determinants that drive them, but also in identify common and emerging themes for their revalidation and reconceptualisation respectively when data are analysed at the global level.

### Presentation

#### Provide granular results

For improved usability and impact of global indexes, priority should be placed first on a context-specific interpretation of results (outcomes, dimensions, indicators) to generate real-world, actionable insights in each country and then cross-country comparison. This can be achieved by presenting granular data on national-level contextual factors such as debt risk, foreign investment or assistance level, and externalities risk that directly influence the ability of countries to respond with effective policy and systemic interventions and, in turn, shape the results of global indexes’ dimensions and indicators. These factors could be rated as ‘high,’ ‘medium,’ or ‘low’ through an annual, multistakeholder review and consensus process and then used to disaggregate global indexes to provide some explanations for the results observed at the national, regional, and global levels.

#### Provide comparable ranking

While cross-country ranking appears to still have some benefits if results are accurate, interpreted correctly, and acted upon decisively, efforts should be made to shift away from international ranking to regional and national ranking. This is important as a trade-off to reduce the level of uncertainty inherent in any global index rank as a result of the large differences in the baseline characteristics and contexts between countries. By pursuing a fairly comparable ranking approach, where countries and states are ranked within similar groups (region and country, respectively), the assumption is that this level of uncertainty can be considerably reduced, and there is a unique opportunity for real-world impact. This is because policy and systemic interventions and investments that are determined appropriate for addressing global indexes’ dimensions and indicators and associated inequities are implemented at the subnational and national levels and then strengthened at the regional and global levels. Additionally, given the variability in the definition of the world’s regions between actors, a consensus for a common classification must be made. Further, regionally and nationally ranked global indexes should be disaggregated by a country and state development level (e.g. HICs vs. LMICs or any new grouping) to effectively identify the most poorly performing countries and states and equitably allocate the needed resources for necessary actions.

Overall, to practically apply these principles and best practices, we propose that ‘principles’ (e.g. representativeness) and ‘best practices’ (e.g. engaging underrepresented stakeholders) could be taken as ‘criteria’ and ‘indicators’ of a technical guideline, respectively, for evaluating the appropriateness of global indexes before their standardisation and adoption (**Figure 2**).

## IMPLEMENTATION PRIORITIES

To operationalise and sustain the recommendations for the ethical design and use of more scientifically robust, practical, and equitable global indexes to support countries, particularly LMICs, towards sustainable development, global institutions and political actors such as the UN and the G20 should urgently consider prioritising the following implementation activities by 2025.

### Technical guideline and ethical framework development

Given the lack of common standards for which the innovators/adopters and users of global indexes can be guided scientifically and ethically, as evidenced, for example, by the variability in the conceptualisation, analysis, and presentation of scientific practices for MPI, GHSI, and HDI (**Table 2**), there is an unmet need to develop a technical guideline, including criteria, indicators, and ethical standards to improve the design, use, and impact of global indexes using evidenced-based principles and practices such as those recommended in this paper (**Figure 2**) and others like the linkage of their indicators to SDG indicators as reflected in MPI and HDI [24,36]. This is important for ensuring equity, enhancing accountability, and minimising unintended risks. In addition, this effort should be led by the UN, and in collaboration with academia, national governments, civil societies, and relevant stakeholders.

### Regulatory and coordinating mechanism

There is a need to establish a regulatory and coordinating mechanism using a whole-of-government and whole-of-society approach to reduce the biased design, fragmented use, and redundant impact of global indexes. Having such a governance mechanism in place is important to ensure the compliance of existing and future global indexes to the established standards and to develop an integrated architecture for their use such that they are concurrently deployed through national surveys within comparable time points and their results are summarised in a single report. This mechanism must be guided by participatory and collaborative governance, equity, and transparency principles. For example, the Academic Council on the United Nations System provides a suitable platform that can be leveraged to achieve this because it is an institution with a global network of stakeholders, including professionals, policymakers, and populations across multiple sectors, disciplines, and specialities working to address issues of international concern, including global governance and sustainable development [50]. A leadership structure, including a steering committee providing oversight and strategic guidance, a technical advisory group making expert reviews and recommendations, and a facilitating group supporting global indexes guideline development, curation, evaluation, joint deployment, and harmonised reporting should be created to implement planned actions effectively, efficiently, and sustainably.

### One survey, one budget, and one report

To actualise the proposed integrated architecture for the joint deployment and harmonised reporting of global indexes, one survey, one budget, and one report approach should be considered. By having one survey collected electronically through national surveys, issues with differences in the methods and time points of data collection can be easily resolved. Additionally, necessary data elements can be shared with all relevant stakeholders in real-time seamlessly, safely, and securely leveraging emerging digital technologies (e.g. blockchain, cloud, application programming interface) built on strong data privacy, exchange, and data security frameworks. Blockchain provides the benefits of ensuring that data are time-recorded and immutable (i.e. data value can’t be changed once recorded) with data transparency, integrity, and auditing enhanced, while the cloud makes it easier to collaborate and automatic data backup [51–53]. By maximising the benefits of blockchain and cloud with an application programming interface based on a set of predefined data exchange, data analysis, and data storage rules to build an integrated, decentralised architecture for data storage and management, the challenges of data manipulation and limited remote collaboration for data analysis, and loss of data can be greatly addressed [52,53]. More so, it will help to reduce the burden of research participation among the population. Likewise, with one budget through a pooled funding system, data collection, analysis, and reporting costs are substantially reduced, and saved costs can be invested in other equally important policy areas. In addition to the comprehensive, individual report on global indexes, having one report on all relevant global indexes at the national level with concise yet detailed information could help improve policymaking and optimise resource mobilisation and allocation. This is because the results of all global indexes can be evaluated concurrently with those of national surveys to have a clearer picture of issues, which is important for identifying priorities, designing interventions, informing budgeting, implementing intervention activities, and evaluating them adequately.

### Inequity and impact evaluation

With only five years left to achieve the SDGs [33], there is a need to advance the value of global indexes to accelerate progress. Combining insights from context-specific results and comparable cross-country ranking of global indexes with inequity and impact evaluation gives a crucial opportunity to provide the right mix of policy and systemic interventions and investments. Understanding the wider structural determinants, including political, economic, and social systems in which inequalities are created, ineffective policies are shaped, and inequities are sustained, is vital for systemic reforms in addressing them to improve people's lives and protect the planetary ecosystem towards sustainable development. To better understand the root causes of inequity in global indexes, associations between political systems (e.g. centralised or decentralised), economic systems (capitalist or socialist), and social systems (caste or class) and global indexes can be assessed through linear regression analysis across countries [54–58]. And, to also evaluate the policy impact of global indexes, a correlational analysis can be conducted to confirm a strong-moderate positive relationship between absolute change in global indexes and absolute change in current expenditure on relevant outcomes over a given period (e.g. GHSI vs. current health expenditure, MPI vs. current health, education, and social protection expenditure). As a forward-thinking approach, countries can be grouped into categories with similar wider structural determinants, which can then form the basis for a within-group ranking system (e.g. regional ranking) to improve cross-country comparison.

## CONCLUSIONS

We live in a time of unprecedented global issues that threaten the lives of people and the balance of the planetary ecosystem in complex and dynamic ways. With SDGs in sight for achievement by 2030 and countries’ progress evaluated in part by numerous global indexes that have been found to have biased designs, fragmented use, and redundant impact with limited ethical considerations [15–17,21,33–35,59], there is an urgent and critical need to rethink ‘who’ and ‘what’ determines what is measured about our world, and ‘how’ these tools are developed and applied with ethical considerations if we must meet this target. It is time for all stakeholders, including the UN agencies, national governments, academia, donors, businesses, NGOs, and the general public, to advance the fields of measurement science and data science to promote the ethical design and use of more scientifically robust, practical, and equitable global indexes.

A paradigm shift is needed to move the ideologies and practices in the conceptualisation, analysis, and presentation of global indexes from an era of ‘what can be measured based on available data for cross-country ranking’ to ‘what should be measured based on data made available for context-specific results with comparable cross-country ranking.’ Building on gains and lessons learned from global issues (e.g. COVID-19, international cooperation) and leveraging scientific and organisational best practices, existing resources, and emerging digital technologies harmonised through a whole-of-society approach, there is no better time than now for the global community to authentically collaborate to enhance the methodology of global indexes for equitable evidence-informed policy in improving people’s lives and strengthening national capacities towards sustainable development. Finally, a culture of community envisioning, ongoing deliberation, and learning through transdisciplinary thinking must be pursued to maximise impact.
